# Intranasal Dental Repulsion of a Displaced Cheek Tooth in an Arabian Filly

**DOI:** 10.3390/ani15060772

**Published:** 2025-03-08

**Authors:** Alessandro Spadari, Giuditta Saragoni, Federica Meistro, Maria Virginia Ralletti, Francesca Marzari, Riccardo Rinnovati

**Affiliations:** Department of Veterinary Medical Sciences, University of Bologna, Via Tolara di Sopra 50, 40064 Ozzano dell’Emilia, Italy; alessandro.spadari@unibo.it (A.S.); giuditta.saragoni2@unibo.it (G.S.); federica.meistro@unibo.it (F.M.); francesca.marzari2@unibo.it (F.M.); riccardo.rinnovati2@unibo.it (R.R.)

**Keywords:** equine cheek tooth, dental disease, cheek tooth displacement, intranasal repulsion, tooth, horse

## Abstract

This case report documents an unusual cheek tooth (CT) displacement in a 3-year-old Arabian filly. The horse was referred to the clinic for loss of appetite and right-sided facial deformity. At admission, an oral inspection was performed, followed by a dental radiographic examination. The radiographs showed a very oblique vertical angulation (135°) of the CT 106 compared to the other premolars (107 and 108 CTs) (40°). In consideration of the clinical and radiographic findings, surgical extraction of the 106 CT was indicated. Due to the unique positioning of the displaced tooth, traditional extraction methods such as standing oral extraction or minimally invasive buccotomy were deemed impractical. An innovative intranasal dental repulsion technique was selected to minimize invasiveness while ensuring the possibility of performing a complete oral extraction. The procedure was performed under general anesthesia without requiring skin incisions or a lateral osteotomy of the maxilla. The surgery was successful, with no intra-operative or post-operative complications. This original approach offers a viable alternative for similar cases, reducing the morbidity associated with conventional repulsion techniques.

## Simple Summary

Dental diseases in horses, particularly those associated with disorders of the cheek teeth, are frequently encountered and can significantly affect an animal’s overall health. Consequently, equine dentistry has advanced rapidly in recent decades, introducing innovative techniques that enable veterinarians to conduct most exodontia procedures using only standing sedation. General anesthesia is now primarily reserved for more complex cases. This case report presents an unusual dental disease along with an alternative surgical approach for its extraction, due to the tooth’s unusual inclination. The methods of tooth extraction continue to evolve, aiming to create increasingly minimally invasive and less traumatic surgical procedures. Further studies and additional cases treated with the described technique warrant consideration.

## 1. Introduction

Dental diseases are among the most significant oral pathologies in horses, and therefore in equine veterinary practice [1]. Equine dentistry has progressed rapidly in recent decades, with advancements in diagnostic imaging and extraction techniques [2,3]. Dental disorders in horses can be of common occurrence and can cause significant health problems [4]. Cheek teeth disorders are a common cause of morbidity, and in severe cases can lead to life-threatening conditions for the animal [5]. Horses have hypsodont teeth characterized by long crowns. The short length of the crown that is visible within the mouth is called the ‘clinical crown’, and the longer un-erupted portion is the ‘reserve crown’ [6,7]. Dental eruption occurs up to the age of twenty [7,8], but slows as the horse ages. The deciduous teeth shed in a well-described order, becoming known as “caps” as they approach the time for replacement. In some cases, these tooth remnants may be retained, causing discomfort and requiring extraction [6,7,9]. In the presented case, the target tooth is the CT 106. Focusing on some anatomical features of this specific tooth, it is known that this is usually the shortest one (5–6 cm maximum length in Thoroughbreds), with the remaining CT up to 10 cm at eruption in Thoroughbred-sized horses [9]. Normally, the angle of incidence ranges from approximately 160° to 180° in young horses, decreasing to less than a right angle as the incisors exhibit a forward and outward slant with age. As this slant increases, the surfaces of the lower corner teeth do not wear down completely to the posterior margin of the upper incisors, resulting in the formation of a dovetail, notch or hook at the upper corners by the age of seven years [10]. In particular, the long axis of a CT is commonly quite straight, except for the CT 106, whose clinical crowns tilt caudally and whose reserve crowns tilt rostrally. The purpose of these angulations is to compress all six cheek teeth tightly together at the occlusal surface (masticatory surface) to prevent developmental problems [9]. Developmental and eruption disorders of the cheek teeth are various and may predispose affected horses to dental impactions, diastemata and malocclusion. Among these, developmental cheek tooth displacement is commonly reported in young horses due to overcrowding or abnormal orientation during eruption [1,11]. Extraction/exodontia remains the treatment of choice for most dental diseases [5]. While repulsion under general anesthesia via large trephines or even sinus flap osteotomies were the mainstay extraction techniques for many years [12], in most cases, these approaches have been superseded by standing extraction techniques thanks to advances in sedation protocols, regional anesthetic techniques, surgical methods and enhanced equipment [1]. Today, a wide range of techniques are available, and can be tailored to the individual circumstances of each tooth to achieve an optimal outcome regarding the morbidity, function, cosmesis and cost [5]. Full osteotomy or traditional buccotomy is only required in rare cases [2,5]. In addition, more innovative and conservative techniques like endodontic and restorative dentistry are developing, taking cues from their benefits in human dentistry [2]. Cheek tooth extraction in horses can be challenging and carries a significant risk for intra- and post-operative complications [13]. In this case, the displacement of the CT 106 resulted in an aberrant angulation that precluded the efficacy of conventional extraction techniques. The aim of the present report is to describe the technique and the outcome of an innovative intranasal repulsion approach for the extraction of an unusually displaced CT 106 in a 3-year-old Arabian filly.

## 2. Case Description

### 2.1. Clinical Presentation

A 3-year-old Arabian filly was referred to the Equine Surgery Unit, Department of Veterinary Medicine, Bologna University, presenting with a loss of appetite and right-sided facial deformity. A clinical examination did not identify any significant abnormalities. Firstly, an extra-oral exam to assess the horse’s facial symmetry and facial muscles was performed. A right-sided distortion of the maxillary bones with facial asymmetry was present, with firm bony enlargements on the ventral margin of the horizontal ramus of the mandible. Palpation of the maxillary swelling elicited mild discomfort, though no nasal discharge or submandibular lymphadenopathy were noted. Sedation with detomidine hydrochloride (0.01 mg/kg) enabled a thorough intra-oral exam, revealing remnants of the 506 tooth and a palpable, firm and slightly painful bulge in the right ventral nasal diverticulum arising from the lateral part of the nasal process of the incisive bone, almost at the level of the synarthrodial surface of the maxillary bone, suggesting abnormal positioning of the underlying CT 106. No signs of malocclusion or oral soft tissue alteration were noticeable.

### 2.2. Diagnostic Imaging

**Figure 1:**
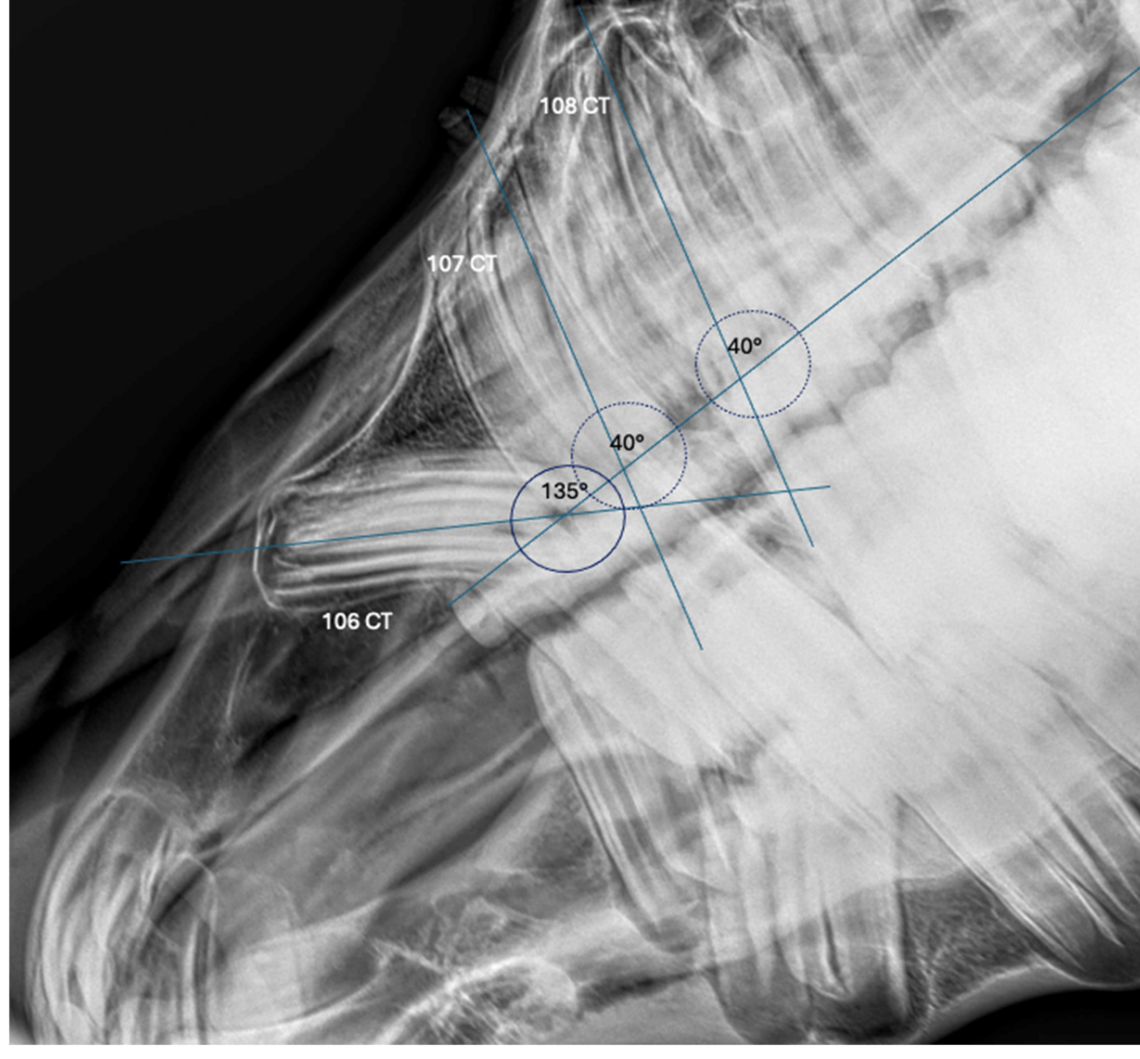
A radiographic cheek teeth study: one longitudinal line passing through the occlusal surface and one line passing through the major axis of each premolar were traced. The alignment angles for each tooth were calculated. The CT 106 showed an angulation of 135°, compared to the 40° angulation of the CT 107 and 108. In addition, there was impingement with the rostral margin of the clinical crown of the CT 107. The oblique lateral view revealed a smooth-bordered periapical lucency and cortical bony enlargement involving the 406, 407 and 408, justifying the irregular ventral mandibular profile.

Radiographic evaluation included laterolateral, dorso-ventral and latero-30° dorsal-latero-ventral-oblique views confirming an abnormal angulation of the 106 CT. No alterations were shown either in length or in the radiographic pattern of the tooth compared to its corresponding contralateral CT. A study on the radiographic image to determine the angulation of the CT 106 was conducted. One longitudinal line passing through the occlusal surface and one line passing through the major axis of each premolar were traced. The alignment angles for each tooth were then calculated. The CT 106 showed an angulation of 135°, compared to the 40° angulation of the CT 107 and 108. In addition, there was impingement with the rostral margin of the clinical crown of the CT 107. The oblique lateral view revealed a smooth-bordered periapical radiolucency and cortical bony enlargement involving the 406, 407 and 408, justifying the irregular ventral mandibular profile (Figure 1). Finally, a diagnosis of the unusual developmental displacement of the CT 106 was made. From the clinical inspection and the radiographic images, it seemed that the tooth apex lay close to the lateral part of the nasal process of the incisive bone. However, the main concern was that the tooth crown actually resided within the maxillary bone and protruded towards the incisive bone and nasal passages at its apical end, causing the pain. Another point of concern was that the source of pain was a potential periapical infection (even if radiographically silent), as is commonly seen with vertical cheek teeth impactions. Given the observed dental displacement and the clinical and radiographic signs, an extraction of the CT 106 was considered the most appropriate therapeutic choice. A CT scan was considered, but was unavailable at the facility, and the owner declined a referral to another CT scan-equipped clinic.

### 2.3. Surgery

#### 2.3.1. Surgical Planning

The selection of an appropriate extraction technique was influenced by multiple factors, including the horse’s age and temperament and the displaced tooth’s position. Standing oral extraction was ruled out due to the extreme angulation and partial eruption with the absence of the clinical crown of the CT 106, making vertical extraction unfeasible. For these reasons, general anesthesia was deemed necessary. Maxillary osteotomy was left as the last choice due to its invasiveness and high rate of complications. Minimally invasive buccotomy and screw extraction was also considered. The fact that the bulging was easily palpable from the nasal diverticulum led the surgeon to think about a new approach with a direct repulsion on the suspected apex of the tooth, not requiring any osteotomy or skin incision. In this case, the complication rate associated with repulsion could be reduced due to the absence of osteotomy. Conclusively, it was decided to perform an intranasal cheek tooth repulsion under general anesthesia.

#### 2.3.2. Preparation of the Patient and Surgical Procedure

**Figure 2:**
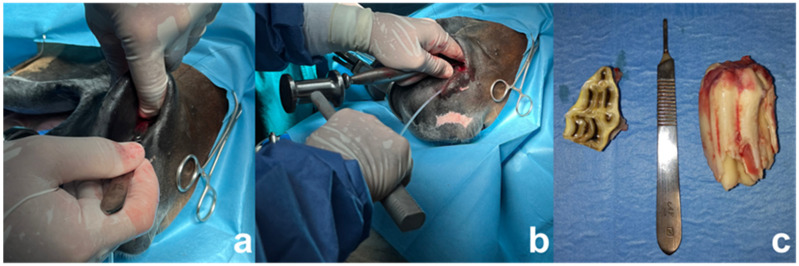
Intranasal surgical approach: (**a**) intranasal surgical access; (**b**) retropulsion of 106 CT with dental punch positioned through nasal cavity; (**c**) from left to right: capsule and extracted 106 CT.

The surgery was performed under general anesthesia with the horse in lateral recumbency. The horse received preemptive analgesic treatment with intravenous flunixin meglumine (1.1 mg/kg). Sedation was administered using romifidine hydrochloride (80 microg/kg). Perineural local anesthesia of the right maxillary nerve was performed using 5 mL of lidocaine 2%, as described by Stauffer [8]. General anesthesia was induced with ketamine (2.5 mg/kg) and diazepam (0.05 mg/kg). Maintenance was obtained with isoflurane (iso-MAC value 1.20%) administered with an orally inserted endotracheal tube, in order not to interfere with the intranasal surgical procedure. A sterile catheter was inserted into the nasolacrimal duct to aid visualization and prevent inadvertent trauma during the surgical manipulation. A flat-bladed elevator was used to separate the buccal and palatal gingiva from the affected crown. First, the CT 106 cap was removed. Then, an intra-oral dental luxation of the CT 106 clinical crown was performed using a molar spreader. The molar spreader was delicately placed distally to the target tooth, and slow, steady pressure was applied to stress the periodontal tissue while intranasally feeling the movement of the apex. At this point, an incision of the nasal diverticular mucosae was made in correspondence to the protuberance, which was perceivable to be the CT 106 apex. Using molar extraction forceps orally, the tooth was grasped and small horizontal movements were made, gradually increasing the amplitude as more instability was appreciated in the tooth. The CT 106 was consequently partially luxated from its alveolar ligament, making it even more mobile and therefore easily removable. A dental punch was positioned intranasally on the tooth apex, following the eruption pathway of the tooth. A bone mallet was used on the punch to repulse the tooth. Despite the tooth orientation, the technique resulted effectively in pushing the CT within the oral cavity. With the tooth partially repulsed in the oral cavity, it was then possible to grasp it with the molar extraction forceps and get the right angulation to extract the tooth with only a few intense movements (Figure 2). A curettage of the alveolus was performed. No osteotomy or maxillary bone damage was made. Briefly after the extraction, the alveolar cavity was irrigated with saline. An intra-operative radiograph was performed to confirm the complete dental extraction and to evaluate the eventual damage to the surrounding structures. The nasal mucosa overlaying the alveolus was closed in a simple interrupted pattern using 2-0 USP BIOSYN™ synthetic absorbable sutures. A plaster of Paris was positioned within the alveolus to avoid food pocketing. It was checked that the pack would fill no more than one-third of the alveolus to avoid hindering the granulation tissue formation and healing of the defect.

### 2.4. Post-Operative Management

The horse’s recovery from general anesthesia was uneventful. Post-operative analgesia was provided with flunixin meglumine (1.1 mg/kg IV for four days), and the antimicrobial therapy included gentamicin sulfate (6.6 mg/kg IV) and penicillin–streptomycin (9000 IU benzylpenicillin + 11.3 mg/kg dihydrostreptomycin IM for five days). The intra-oral surgical site was inspected under sedation two days post-extraction to check if the plaster package was still in place, and no complications to the healing process were reported. After seven days, the plaster of Paris was removed to better examine the alveolus, which was already partially filled with healthy granulation tissue. The hole created by the repulsion could still be felt within the alveolus, but with a resulting reduction in size. The alveolus was disinfected with 4% chlorhexidine solution diluted with water and left without the plaster filling. Also, the healing process of the nasal diverticular incision was healthy, without exuberant scar tissue. The alveolus was additionally checked one day before the horse was discharged. The filly was hospitalized for ten total days. No further therapies were prescribed.

### 2.5. Long-Term Follow-Up

**Figure 3:**
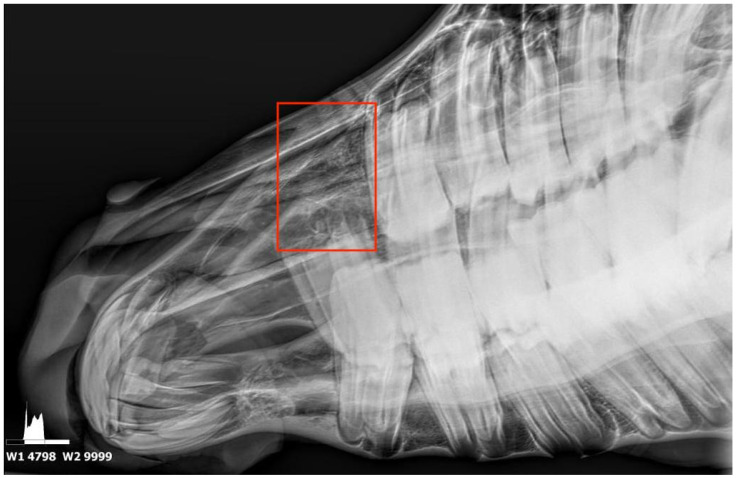
Four-month post-operative lateral oblique dental radiograph showing no bone or soft tissue alterations. The red rectangles shows the area where the displaced 106 CT was positioned before extraction.

A follow-up phone call was made to the owner one month after the filly’s discharge, which provided the information that no complications were present. Four months later, the referring veterinarian took follow-up radiographs, which revealed no abnormalities (Figure 3); he also reported that the bulging in the nasal cavity was no longer perceptible at palpation. The outcome remained unchanged at a 9-month phone call follow-up.

## 3. Discussion

The present report represents the first documented case of an intranasal dental repulsion of an abnormal displaced CT 106 in a horse. Two different kinds of displacement are described in horses: the developmental and the acquired. The developmental type of displacement tends to affect younger horses, and it usually concerns the caudal CTs, like the 4th maxillary and 5th mandibular CTs [14]. Also, the permanent 06, 07 and 08 are seldom affected with overcrowding or developmental displacements because they replace deciduous teeth of the same size [15]. This kind of displacement is usually bilateral, and rotation of the affected tooth is occasionally present, but not in this case [14]. Dixon et al. reported also that 70% of cheek tooth displacements were developmental in origin due to overcrowding of the cheek tooth row at the time of eruption. The remaining 30% of displacements were felt to be due to abnormal positioning of the CT buds, which may result in non-eruptions of the displaced teeth if they are sufficiently horizontal to the adjacent teeth [14,15]. Trauma to the jaws can also cause damage or displacement of the CT buds, causing later mal-eruptions [15]. On the other hand, acquired CT displacements (usually medial displacements of the lower 10 s and 11 s) frequently develop in older horses. The exact cause of these acquired CT displacements is unclear. In some cases, the acquired displacements may be favored by abnormal angulation of the CT occlusal surfaces (e.g., shearmouth). Another possible cause of displacement is pre-existing periodontal disease, which can reduce support against the normal masticatory forces [1]. Various cases of displaced maxillary cheek teeth have been reported in the literature. Colyer described three cases of maxillary CT displacement with rotation of the 2nd and 3rd CTs, but he noted that these kinds of displacements most commonly affected the caudal CTs [16]. Two cases of palatal displacement of the 3rd maxillary CT were described by Cook and Baker [17,18]. Wafa illustrated that CT displacements most commonly affect the 3rd and 4th CTs, in some cases bilaterally, both in the upper and lower arcade. Becker and Edwards reported cases of non-erupted displaced CTs that lay horizontally in the mandibles or maxillae [19,20]. The most similar case to the one reported in this article is the one described by Robert et al. in which the horse showed symmetrical bilateral swelling due to a displacement of the 107 and 207, with an important caudal inclination of the 106 CT [21]. In this case, exodontia of the CT 106 seemed to be the most appropriate therapeutic choice. Cheek tooth extraction in horses is indicated for many conditions [13], even if it may present many challenges for even the most experienced clinicians [4]. In the majority of cases, dental extraction is carried out using a standing procedure with the horse sedated. A small percentage of horses with select pathologies are put under general anesthesia to perform cheek teeth extractions [2,4,5,22,23]. The techniques that are commonly used include standing oral extraction with or without partial coronectomy [2,23], oroscopically guided fragment extraction [24], Steinmann pin repulsion, and minimally invasive trans-buccal surgery and intradental screw placement (MITSE), which are used in cases where failures occur during conventional extraction attempts [25], up to the use of more innovative techniques that can prevent exodontia, including restoration techniques for infundibular caries [26] and endodontic therapies that have been used, for example, in the treatment of acute exposure of the vital pulp [27]. In many studies, intra-oral extraction in a standing sedated horse is the optimal exodontic technique where a sufficient clinical crown remains. For this reason, this approach should be considered the gold standard [12,13,23,28,29]. Full osteotomy, buccotomy and traditional large punch repulsion are generally considered obsolete techniques that are used only in select cases. Significant mechanical force is required for exodontia due to the great length of equine cheek teeth reserve crowns (>9 cm long in younger horses). Damage to the alveolar and supporting maxillary and mandibular bones is possible [30]. For this reason, in horses, CT extraction can be challenging and has a significant risk of intra- and post-operative sequelae. Some frequent post-extraction complications include delayed alveolar healing, including sequestration of the alveolar bone with alveolar infection, osteomyelitis of the supporting bones, retention of dental fragments, persistent dental sinusitis, damage to adjacent teeth, adjacent soft tissue or bone damage, and formation of oronasal, oro-maxillary or oro-cutaneous fistulae [13,15,22,23,28,31]. Post-operative problems have been reported in 23–80% of horses following cheek tooth repulsion under general anesthesia, with the highest levels following maxillary cheek teeth extractions [12,13,29].

Standard repulsions in standing horses caused post-extraction problems in 41% of cases [31]. The lateral buccotomy technique recorded post-extraction problems in 27–53% cases. Additional associated risks are general anesthesia and the possibility of neurovascular and parotid duct damage [13,30]. Oral extractions have a lower post-extraction complication rate (3.6–20%) compared with repulsion or lateral buccotomy techniques [12,13,23,28]. In this case, the positioning of the displaced cheek tooth influenced the authors’ surgical approach. In fact, considering the age of the animal, its anxious temperament and the position of the tooth, an intranasal procedure under general anesthesia was chosen. More aggressive techniques that include a more invasive maxillary osteotomy are considered a choice of last resort, where every other technique has failed. An intranasal repulsion of the tooth was studied and implemented, mainly because the bulging corresponding to the suspected apex of the CT 106 was easily palpable from the nasal diverticulum, allowing the surgeon to put the dental punch directly on the tooth apex. It would have been very complicated to perform this procedure on a standing horse, considering that intranasal repulsion, even with a good standing sedation protocol, can increase the risk of the patient moving during the surgery, leading to possible extensive hemorrhage, lacrimal duct lesions and impossibility of obtaining the correct and precise angulation for the effective repulsion of the tooth, in addition to a longer surgery time. The main advantage of this specific extraction technique turned out to be that a cutaneous incision and lateral, more invasive osteotomy was avoided. In addition, the technique guaranteed an optimal aesthetic result, since the mucosal incision was made through the nasal diverticulum. In the present case, no intra- or post-operative complications were reported, confirming the effectiveness of the technique.

## 4. Conclusions

To the best of the authors’ knowledge, this is the first report detailing a surgical intranasal repulsion of a displaced cheek tooth. In the vast majority of cases, alternative extraction techniques will be more appropriate, but this approach should be considered a valuable alternative in the very small subset of cases where the 106 or 206 cheek teeth are displaced and angled with their apices lying rostrally adjacent to the nasal passages. Extraction methods continue to evolve, with the purpose of designing more minimally invasive and minimally traumatic surgical procedures; considering the absence of intra- and post-operative complications, the described technique should be considered as a valid alternative for the treatment of dental pathologies, and in particular for developmentally displaced cheek teeth. Further studies and cases treated with intranasal repulsion should be taken into account.

## Data Availability

No new data were created or analyzed in this study. Data sharing is not applicable to this article.
